# Climate-Conscious Glaucoma Care: Strategies to Minimize the Environmental Impact in the Operating Room and in the Clinic

**DOI:** 10.1155/joph/7188835

**Published:** 2025-07-10

**Authors:** Samantha R. Goldburg, Lucy Li, Emily Schehlein, Aakriti G. Shukla, Mary Qiu

**Affiliations:** ^1^Department of Ophthalmology, Northwell Health, New Hyde Park, New York, USA; ^2^Department of Ophthalmology, Zucker School of Medicine at Hofstra-Northwell, Hempstead, New York, USA; ^3^Department of Ophthalmology, New York Medical College, Valhalla, New York, USA; ^4^Brighton Vision Center, Brighton, Michigan, USA; ^5^Department of Ophthalmology, Edward S. Harkness Eye Institute, Columbia University Irving Medical Center, New York, New York, USA; ^6^Department of Ophthalmology, Cole Eye Institute, Cleveland Clinic, Cleveland, Ohio, USA

## Abstract

The healthcare sector contributes significantly to greenhouse gas emissions and global warming. There is an increasing prevalence of glaucoma, and glaucoma surgeries, nonsurgical treatment, and clinic follow-up contribute to these emissions. Some of the main sources of emissions associated with glaucoma care are related to transportation to and from surgical centers and clinic, single use equipment and eye drops, excessive device packaging, and waste produced in the operating room. There are several changes we can make to our practice patterns to help mitigate these emissions while maintaining safe and effective care for our glaucoma patients. We should emphasize disinfecting equipment properly rather than purchasing single-use items. We should perform procedures that utilize equipment that is locally available and recommend manufacturers to use smaller packages for glaucoma devices. We should strive to perform bilateral procedures when safe for patients. Finally, we should integrate telehealth into our regular practice.

## 1. Introduction

The number of people affected by glaucoma worldwide continues to increase and is estimated to be nearly 112 million by 2040 [1]. As glaucoma prevalence rises, there will be a growing number of people who require timely diagnosis and management of this potentially blinding condition. Resources essential to providing this care will further challenge already stretched healthcare systems and will contribute to the impending climate crisis.

Human activity, particularly the combustion of fossil fuels, causes warming of the atmosphere, land, and oceans, leading to climate change [2]. In turn, planet Earth is experiencing heat waves, resulting in increased health risks [2, 3]. Moreover, vector-borne diseases are expected to increase over the next 80 years if mitigation efforts are not taken to control global warming [4]. These health effects are predicted to affect marginalized racial and ethnic groups and low-income populations to a greater degree, due to limited access to care and resources to combat the heat, more frequent occupational exposures, and higher incidence of chronic medical conditions [2, 5].

The healthcare sector accounts for about 10% of greenhouse gas emissions in the United States (US), with the operating room (OR) as one of the biggest sources of medical waste [6]. The field of ophthalmology contributes significantly to these emissions, due to the high volume of surgical procedures utilizing disposable materials [7]. An estimated 5.2 million cataract surgeries were performed in 2017 in the United States, and this rate is expected to grow 72% by the year 2036 [8]. The number of reported cataract surgeries globally differs across various studies and ranges from 36 per million in Malaysia to 12,800 per million in Sweden [9]. The number of glaucoma surgeries increases by 10.6% from 2008 to 2016 [10], with an explosion of novel procedures in the minimally invasive glaucoma surgery (MIGS) category. MIGS increased by 426% from 2012–2016 in the United States [10] and continued to become more common alongside the interventional glaucoma movement, which suggests that glaucoma procedures should be performed earlier in the disease course [11].

Ophthalmologists, environmental scientists, and others have performed lifecycle assessments (LCA) of ophthalmic surgery to calculate the carbon footprint associated with surgery. An LCA incorporates emissions of a product or process from raw material extraction through use and disposal [12]. In ocular surgery, this takes account into travel of staff and patients, the flow of materials through the OR, the weight of each surgical instrument, sterilization processes, waste based on type of material, waste disposal routes, water and electricity usage, and more [12]. While it may not be feasible to perform a LCA for every ophthalmic surgery, these calculations have underscored the importance of monitoring and ultimately decreasing emissions from surgical procedures.

As the proportion of our aging population grows, glaucoma care will occupy a larger portion of the healthcare sector and will contribute to the climate health crisis that will affect generations to come. This literature review aims to provide background and actionable items to reduce the carbon footprint of glaucoma care. The scope of this assessment includes waste derived from surgery, preoperative and postoperative care, pharmaceuticals, and the office setting.

## 2. Materials and Methods

The present study is a literature review. A search was conducted in the PubMed database to identify relevant peer-reviewed articles. The search included the terms “sustainability AND glaucoma,” “sustainability AND glaucoma surgery,” “carbon footprint AND glaucoma,” “carbon footprint AND glaucoma surgery,” and “carbon emissions AND glaucoma treatment,” with no date or language restrictions. References of included articles were screened manually for additional sources.

## 3. Main Text

### 3.1. Clinic

First-line management for glaucoma includes topical pharmaceutical therapy or selective laser trabeculoplasty (SLT) [13]. A comparison of medications vs. SLT first in patients with a new diagnosis of open-angle glaucoma or ocular hypertension found a similar carbon footprint in both groups [14] when using a treatment schedule as described by the LiGHT trial [15]. However, when the 2-week visit following SLT was removed, as this visit is no longer performed in most cases, the laser group had a lower carbon footprint [14]. The LiGHT trial outcomes suggest that patients who receive laser first have reduced incisional surgeries [13] and therefore may be associated with reduced emissions if additional visits can be minimized. It was found that patients treated with drops first had fewer clinic visits, more medications, and fewer laser treatments, but more cataract and trabeculectomy surgeries [14]. This study emphasizes how significant transportation is to total carbon emissions. Interestingly, the energy used for SLT was found to be negligible in terms of carbon emissions [14]. While emissions from long-term eye drop use and disposable clinic materials were an order of magnitude less than travel emissions [14], they may still have an impact over the patients' lifetimes. A study quantifying the waste generated by laser cosmetic surgery in the United Kingdom also found that the biggest contributor to carbon footprint was staff and patient travel. The carbon emissions generated from the lasers themselves were minimal [16]. In terms of glaucoma treatment, these findings may be extrapolated to other laser procedures beyond SLT. For instance, in patients who need a laser peripheral iridotomy (LPI) or laser suture lysis (LSL), the transportation would like to contribute more to the carbon footprint than the procedures themselves.

Many patients with glaucoma are on several medications. Studies have shown that patient transportation to the pharmacy is responsible for a large percentage of the carbon footprint associated with pharmaceuticals [17]. As prescribers, if we can request that pharmacists release multiple refills at once, and if we can use combination drops rather than individual medications, this may help further decrease patient transportation and therefore decrease the carbon footprint associated with topical glaucoma therapy. Moreover, single-use eye drop bottles have been shown to produce more plastic and paper waste compared to multidose bottles [18].

Single-use equipment has become the mainstay in most ophthalmology clinics given the theoretical risk of disease transmission. In glaucoma practice, tonometer tips and gonioprisms are frequently used, and viral infections of the eye have been linked to inadequately disinfected tonometry tips with 70% isopropyl alcohol (IPA) alone [19]. There are clear AAO and CDC guidelines stating that reusable tonometry tips can be sufficiently disinfected with 5000 parts per million sodium hypochlorite [19]. There are also data showing that using 70% IPA wipes may lead to fine surface scratches on the PMMA material of reusable tips, but these were not clinically meaningful [20]. Therefore, 70% IPA wipes should be recommended as a means of cleaning tonometer tips, as they are just as safe and more cost-effective than soaking tips in a bath of 70% IPA [20]. Moreover, when single-use tonometry tips or gonioprisms are utilized, users may touch them, which nullifies the supposed decreased infection risk [19]. Lockington et al. found that bacteria can be transferred to disposable tonometer holders through physicians' hands even after handwashing [21]. They suggest that disposable holders should be cleaned with alcohol wipes just like traditional Goldmann tonometers, which defeats the purpose of having disposable tips [21]. Furthermore, a systemic review compared three methods of tonometer disinfection: alcohol swabs, hydrogen peroxide, and disposable tonometer tips, in the prevention of nosocomial EKC [22]. The cost per case of EKC averted with peroxide bleaching compared to alcohol swab was $12,152 per case, and the cost per case averted when using a disposable tonometer tip was compared to alcohol swabs was $61,324 [22]. Given that EKC is self-limited and only about 10% of patients have complications (e.g., subepithelial infiltrates), alcohol swabs remain the most logical method for cleaning tonometer tips [22]. Our goal should be to properly disinfect multiuse items in order to prevent patient infection while reducing the carbon footprint of our practice.

### 3.2. Clinic Transportation

Transportation to and from the ophthalmology clinic has been shown to be the greatest source of carbon emissions related to ophthalmic care [14, 23], and it is particularly impactful in more rural areas [14]. Most emissions come from transportation in cars. In one study, cars represented 33% of the patient's travel method, but 95% of the total carbon footprint of patient travel [23]. The most impactful way of decreasing emissions from transportation of patients with glaucoma who require frequent visits would be to decrease travel in personal cars. Patients and employees should travel to the closest site and use public transportation when possible. Another solution would be to incorporate more telehealth into glaucoma care. This leads to a different category of emissions in the form of energy consumption; however, these are minor in comparison to emissions from travel [24].

Several studies have shown high adherence rates and patient satisfaction associated with virtual glaucoma appointments [25, 26]. In one study, 11,034 teleglaucoma consults were made over 3 years and 99.8% of visits were carried out [25]. Another study found no clinically significant changes in VA, IOP, or visual field performance during follow-up of patients who were followed virtually for ocular HTN, glaucoma suspects, or early glaucoma [26]. Virtual visits are most successful when done by glaucoma specialists and if moderate-to-severe or unstable glaucoma patients are excluded [27]. There are many new visual field devices that can be used at home that have shown successful uptake, compliance, and performance among patients, including exams on an iPad tablet [28], a head mounted display [29], a PC monitor or virtual reality glasses [30]. In one study of 20 glaucoma and 15 healthy participants using online circular contrast perimetry (OCCP), there were comparable results when the test was performed at clinic and at home [31]. Participants had a positive attitude towards home OCCP, but there was a high dropout rate for 3 month and 6 month at home testing [31]. Moreover, there are now tonometers that patients can use at home, including the iCare HOME: patients' IOP readings have been shown to correlate well with Goldmann applanation [32]. Therefore, it would be feasible for more glaucoma clinics to integrate telehealth into their regular practice, although complete at home glaucoma care remains a challenge, and does not replace clinic monitoring, especially for those with advanced disease. Monitoring IOP at home can be cumbersome for patients, and careful patient selection and education is important to ensure proper device usage [32].

### 3.3. Pharmaceutical Waste

Single use of eye drop bottles, which is common practice in surgical centers and hospitals, is another significant source of waste. In a 2019 study of four surgical centers, almost 50% of all drugs and two-thirds of topical drugs were discarded after single usage during phacoemulsification surgery. The authors estimated that this drug wastage generated 23,000 to 105,000 metric tons of unnecessary CO2eq emissions annually in the United States [33]. Two studies at the University of Utah found that proper reuse of eye drop bottles on multiple patients did not contribute to increased rates of postoperative endophthalmitis (POE), and there was no detection of bottle top or solution contamination via microbiological and videographic analyses [33]. Reusing drops for multiple consecutive patients undergoing surgery in one day, as is done in the Aravind Eye Care System (AECS) in India [34] and supported by four major ophthalmology societies [35], is a practical way to decrease waste. A study by Jensen et al. at Utah Valley Regional Medical Center discusses a policy that was approved and accepted by the Joint Commission for the use of multidose eye drops in multiple patients, which can help guide other institutions to implement a similar program [36]. The Ophthalmic Instrument Cleaning and Sterilization Task Force suggests using topical drugs in multidose containers for multiple patients until the manufacturer's date of expiration label, with proper guidelines [37], which if followed for both glaucoma surgeries and in-office lasers, could save costs and reduce unnecessary usage.

### 3.4. Ophthalmic Surgery

Ocular surgery using protocols found in Western ORs includes many wasteful practices. These include utilization of single-use surgical devices, full-body drapes, excessive packaging material accompanying devices, patient gowning, and more [38]. Surgeons have demonstrated a strong interest in decreasing this unnecessary waste. In a survey study of American and Canadian ophthalmic surgeons and OR nurses with 1300 respondents, 79% said they would prefer reusable instruments and 93% said they wanted product manufacturers to allow surgeons more discretion to reuse products labeled as single use [39]. In another survey study of primarily European cataract surgeons, 99% of respondents shared concern about global warming and 92% felt that OR waste is excessive and should be reduced [40].

Equipment and medications that are designated as “single use” by institutions and manufacturers are significant contributors to waste. In 2022, a multisociety position paper authored by the American Academy of Ophthalmology, the American Glaucoma Society, American Society of Cataract and Refractive Surgery, and the European Society of Cataract and Refractive Surgery facilitated a national checklist revision that permitted eye drop use up to the manufacturer's expiration date, aligning with the product's actual specifications [35]. Many of the sterilization and aseptic protocols used in ophthalmic surgery were made by regulatory agencies that were based off nonophthalmological work and may not apply to ophthalmology [41, 42]. Most regulatory bodies in the United States prohibit the use of immediate use steam sterilization (IUSS) unless it is recommended by the instrument manufacturer [12]. For instance, in 2014, the Centers for Medicare and Medicaid Services declared that IUSS was not to be used for routine surgical instrument sterilization [43]. However, Chang et al. [44] performed a study that supported short-cycle autoclaving of unwrapped instruments for immediate sequential same-day ophthalmic cases when compliant with FDA-approved sterilizers, leading to the development of ophthalmology-specific guidelines on short-cycle sterilization of eye surgical instruments for sequential same-day cases [45].

One of the reasons the reuse of items labeled “single-use” is prohibited in most ophthalmic Western surgical facilities is due to the assumed cross-contamination risk that could lead to POE [42]. Much of the fear of contamination was renewed during the time of COVID-19; a time with significant anxiety is related to the spread of infection. However, the risk of microbial aerosolization with phacoemulsification was found to be insignificant in multiple studies assessing COVID-19 cross-contamination risk [46–51]. Moreover, there are data from AECS that demonstrates the safety of reusable surgical items. In a study in which 3333 samples were collected from different phacoemulsification materials in AECS that were reused between multiple patients, no bacterial or fungal growth was found, and no POE occurred during the 6-week postoperative (post-op) period [34]. Another publication from AECS compared Western OR protocols to AECS's traditional OR protocols. In one group, patients were not gowned, surgical gloves were disinfected but not changed between cases, OR floors were not cleaned between every case, and multiple patients underwent surgery in the same OR. In the other group, each patient was gowned, surgical gloves were changed between cases, OR floors were cleaned between cases, and surgery was performed on one patient at a time in each OR. The infection control measures taken in the second group did not reduce the POE rate [38]. These findings suggest defensive medicine not based on scientific evidence or infectious risk adds to the environmental footprint of each surgical case without mitigating the risk of complications [52].

Some examples of items that are only used once during cataract surgery in the United States and other Western nations include BSS bags, OVDs, IA tips and sleeves, phaco tips and sleeves, cassettes, tubing, cannulas, and syringes. The AECS reuses all these items for multiple patients without sterilization, except for the instruments that contact a patient's eye, which undergo IUSS without wrapping of surgical instruments [41]. AECS was found to have POE rate of 0.04% in 2 million cases using their standard protocols [34], which is identical to the AAO's reported rate of POE in the United States [53]. The POE rate in the AECS dropped further to 0.01% with adoption of routine intracameral moxifloxacin while maintaining their prior protocols [54]. The causative organisms implicated in POE are most often from the patient's skin flora and sometimes from the surgeon's mouth, rather than from organisms on sterilized instruments or other surfaces of the OR [55].

Every item used in the sterile OR setting comes in a package, and most packages contain excessive material that leads to increased waste. In a British study, one phacoemulsification procedure in the United Kingdom was found to generate about 130 kg carbon dioxide equivalent, which is the same as driving a car 310 miles [56] and 20 times more than AECS [6]. In AECS, 2/3 of waste generated during each phacoemulsification is recycled and 1/3 is split between landfill and biomedical waste incineration [12, 57]. It was found that 25% of this waste is packaging and paper directions included with the intraocular lens [12]. In America, we use full body drapes during ocular surgeries, which are thrown away after every case, whereas AECS uses only plastic face drapes [12]. Khor et al. found that 50% of the general waste pool from phacoemulsification surgeries was recyclable [58]. Additionally, Moya et al. conducted a scoping review of global recycling practices in ORs across multiple surgical specialties and identified that up to 74% of OR waste, particularly preoperative waste, is recyclable [59]. However, the implementation of recycling programs is often hindered by regulatory constraints, lack of education, and logistical difficulties [59]. In a study of waste produced during 20 thyroidectomies in the United Kingdom, packaging waste contained 34% plain paper or cardboard, 31% soft plastic film, 26% laminated paper, 7% hard plastic, and 2% metal foil and only a single item had a recycling label. When extrapolated to all thyroidectomies performed in 1 year, the total weight of packaging was 4.2 tons, of which only 31.4 kg would be recyclable [60]. This is translatable to glaucoma and cataract surgery and is an area of feasible change in practice. Targeted recycling initiatives could focus on specific materials like blue wrap and polyethylene terephthalate plastics [59], as well as adding recycling bins to ORs. While the potential for recycling the packaging for glaucoma and cataract surgery exists, successful implementation requires overcoming regulatory and logistical barriers, strategic partnerships with manufacturers, and increasing awareness and education about sustainable practices. It is important for ophthalmologists to work with representative groups and major purchasers, such as hospital networks, to demand reusable items and any necessary single-use items to be made with cheaper, recyclable packaging that is as minimal in size as possible.

### 3.5. Glaucoma Surgery

#### 3.5.1. Conventional Surgery

There is a limited body of literature on the carbon footprint of glaucoma surgery. Namburar et al. [57] investigated the amount of waste generated during glaucoma surgery in the United States compared to AECS, which found that the amount of waste per trabeculectomy at AECS was significantly lower than that of a Baltimore-area hospital, with comparable complication rates. Specifically, the waste at AECS produced during glaucoma surgeries was calculated to be 26% of the US institution [57]. Combined phacoemulsification trabeculectomies performed at AECS produced more waste than standalone trabeculectomies or standalone drainage device surgeries, as would be expected [57]. However, the carbon footprint of combined surgeries is likely lower than separate phacoemulsification and trabeculectomy surgeries, due to the ability to decrease patient transport when surgeries are combined.

When tube shunts are used, multiple additional pieces of equipment with excessive packaging are introduced, which contributes to the waste as discussed above. Each tube, like each intraocular lens used in cataract surgery, comes in a large package many times the size of the device itself. Tube shunts also require patch grafts, which are typically premade scleral or corneal grafts that also come in large packages. Interestingly, Trier et al. [61] found that large packaging in general was associated with increased rates of contact contamination. In order to help remove excess waste from prepackaged grafts during tube shunt surgeries, we can consider autologous patch grafts, such as scleral lamellar grafts [62], or if the patient already had a prior aqueous shunt placed, we can even consider autologous capsular patch grafts for implant revisions, replacements, or new implants [63].

The procurement sector is also a major source of emissions attributable to surgery, accounting for 54% of emissions in one study of cataract surgeries [56]. The majority of emissions within this sector were found to be linked to supply chains for pharmaceuticals and medical equipment. In a study of the carbon footprint of the ophthalmology department at a single institution in France [23], 12% of emissions from the clinic were found to be attributable to pharmaceuticals and medical devices. One reason why emissions related to pharmaceuticals are so significant is because ingredients are often produced in different countries [64]. The emissions related to glaucoma surgeries likely come from similar sources, given the overlap in the type of equipment used and the reliance on many pharmaceuticals. However, different types of glaucoma surgeries may pose different benefits and harms when it comes to their potential footprint.

As we know, trabeculectomies do not require the placement of a device into the eye, which bypasses the issue of waste from excessive packaging of devices altogether. However, pharmaceuticals play an important role in trabeculectomy surgery, given the use of MMC and 5-FU during filtration surgery. We were unable to find any data showing a difference in the possible carbon footprint between MMC and 5-FU, but given that procurement of pharmaceuticals is a large contributor to overall surgical emissions, it is likely that these medications increase the emissions from a trabeculectomy. Disposal of pharmaceuticals also contributes to the carbon footprint as it requires incineration, which releases greenhouse gases, leading to trapped heat and contributing to global warming. The incineration process emits particulate matter, which affects air quality and can have adverse health effects on nearby communities. Moreover, improper handling and disposal of biomedical waste can lead to the contamination of water bodies, soil, and air, posing risks to wildlife and ecosystems [65]. Unfortunately, trabeculectomy surgeries performed without antifibrotic medications are less successful and are not recommended [66, 67], even if their carbon footprint may be smaller than their counterparts.

#### 3.5.2. MIGS

It is also essential to consider MIGS as part of the discussion of the carbon footprint of glaucoma care, as these procedures are becoming increasingly common. Although we could not find studies comparing the carbon footprint of different types of MIGS, we can apply the principles discussed above. Devices that need to be manufactured and packaged individually are associated with more waste, which means greater emissions. Procedures such as gonioscopy-assisted transluminal trabeculotomy and bent ab interno needle goniectomy may be preferable from a carbon footprint standpoint, as they use readily available equipment in the OR [68, 69]. Of note, these techniques have been shown to reduce IOP and medication dependence to an equivalent degree to their commercial counterparts [68–71]. Decreased reliance on medications would lead to a reduction in plastic waste and transportation to the pharmacy, which would decrease the carbon footprint of glaucoma care.

### 3.6. Perioperative Follow-Up

As described above, transportation is a significant contributor to the carbon footprint for glaucoma care. In addition to limiting transportation to the clinic for follow-up, it is important to assess ways we can limit transportation when it comes to laser procedures and incisional surgeries. To this end, we should perform bilateral laser procedures when possible. In a study assessing the safety and efficacy of same-day bilateral 360-degree SLT, patients had a reduction in IOP or a medication reduction in both eyes, with minimal complications [72]. Bilateral same-day LPI has also been shown to be effective and well tolerated [73]. There has also been a trend towards performing post-op day zero visits after cataract surgery, telehealth post-op visits [74], or even removing post-op day one visits altogether for uncomplicated cases. In a randomized trial, Chatziralli et al. [75] found that post-op day one visits can be omitted for uneventful cataract surgery with no significant differences in BCVA or inflammation at day 28 between those who underwent a post-op day one visit versus post-op day 14 visit. There is not as much literature regarding the omission of post-op day one visits for glaucoma procedures. However, one study by Karimi and Lindfield [76] found that for ab interno Xen gel stent surgeries, significant complications requiring intervention on post-op day one were rare. Of the 259 ab interno Xen gel stent cases analyzed, only one case required additional topical IOP lowering medication, and another needed stent manipulation at day one [76]. Therefore, in low-risk scenarios, post-op day zero visits or telehealth post-op day one visits may be acceptable, but for high-risk patients, post-op day one visits should still occur in person. By consolidating follow-up visits, multiple patient trips to the clinic are minimized, thus reducing the environmental impact associated with clinic resource utilization and transportation.

## 4. Conclusion

It is critical for us to change our practice patterns to reduce our carbon footprint during glaucoma surgery and office-based care, while maintaining patient safety and treatment efficacy. Ophthalmologists and other providers can take several steps to move forward on a path towards lower carbon emissions in practice. When it comes to equipment and eye drops, whether in the OR or clinic, the emphasis should be on disinfecting properly rather than purchasing single-use items. Recycling bins should be added to ORs and clinics, and staff should be taught how to use them. We can shift our focus toward procedures that do not require excess procurement and utilize equipment that is locally available. We recommend manufacturers use smaller packages for glaucoma devices without compromising sterility and digitize instructions for use manuals. We can additionally perform bilateral laser procedures and avoid post-op day one visits when possible. Finally, we can integrate telehealth into our regular practice to decrease the significant emissions associated with transportation. These steps will offer the foundation to make impactful change in healthcare waste. Ultimately, we hope to see all subspecialties emphasize environmental stewardship, ideally with a standardized way to measure progress in this area [77].

## Data Availability

Data sharing is not applicable to this article as no datasets were generated or analyzed during the current study.
